# Somnambulism

**Published:** 1834

**Authors:** 


### Art. V.—Somnambulism.

*An account of Jane C. Rider, the Sringfield Somnambulist.* By L. W. Belden, M. D. Springfield, 1834, 18mo., pp. 134.

The object of this neat little narrative, is to give an authentic professional and popular acount of a case, which has lately excited much wonderment among the people, particularly of the eastern states. The author of the narrative, Dr. Belden of Springfield, Massachusetts, has acquitted himself well—that is without running either into credulity or scepticism.

The disorder of Miss Rider was one which cannot be very easily defined. It was not sleep, nor dreaming, nor delirium, nor mania, and still it bore a resemblance to each or all. Dr. B. has applied to it the term of somnambulism, or sleep-walking, and perhaps no better word now *exists*, by which to designate it; but it is with no great propriety that such a state of mind is regarded as a modification of sleep. Because it *generally* came on when she was asleep, it does not follow, that it is a variety of that state. Very often, moreover, it came and went in the day time, without being preceded, attended or followed by the phenomena of sleep. But our object is to lay the facts in the case before our readers—not to speculate upon them.

The patient, 17 years old when affected with this malady, was a native of Vermont. She lost her mother in infancy from a disease of the *brain*. She was taken to Springfield and grew up there. Her intellectual faculties were well developed. From her earliest recollection, a small spot on the left side of her head, near the phrenological region of *marvellousness*, was tender or painful on pressure, and always felt much worse when she had headache, a disease to which she was liable. It was generally attended with a manifest determination of blood to her head. When 14 years old, she had chorea for several months. She was, while growing up, a sound sleeper, but occasionally rose and walked in her sleep. Her eyes, for years, were inordinately sensible to light. At 17 the catamenial function was not yet established.

The first distinctly marked paroxysm of somnambulism, was on the night of the 24th of June, 1833. Dr. B. found her struggling to get out of bed. She complained of pain in the left side of the head, her whole head was hot, her face flushed, her eyes closed and her pulse much excited. She took an emetic, threw up a quantity of undigested currants, and went quietly to sleep. The next morning she knew nothing of what had happened.

"Nearly a month elapsed before another paroxysm. Then, after several attempts to keep her in bed, it was determined to suffer her to take her own course, and watch her movements. Having dressed herself, she went down stairs, and proceeded to make preparations for breakfast. She set the table, arranged the various articles with the utmost precision, went into a dark room and to a closet at the most remote corner, from which she took the coffee cups, placed them on a waiter, turned it sideways to pass through the doors, avoided all intervening obstacles and deposited the whole safely on the table.

She then went into the pantry, the blinds of which were shut, and the door closed after her. She there skimmed the milk, poured the cream into one cup and the milk into another without spilling a drop. She then cut the bread, placed it regularly on the plate, and divided the slices in the middle. In fine, she went through the whole operation of preparing breakfast with as much precision as she could in open day; and this with her eyes closed, and without any light except that of one lamp which was standing in the breakfast room to enable the family to observe her operations. During the whole time, she seemed to take no notice of those around her, unless they purposely stood in her way, or placed chairs or other obstacles before her, when she avoided them, with an expression of impatience at being thus disturbed.

She finally returned voluntarily to bed, and on finding the table arranged for breakfast when she made her appearance in the morning, inquired why she had been suffered to sleep, while another had performed her duty. None of the transactions of the preceding night had left the slightest impression on her mind—a sense of fatigue the following day being the only evidence furnished by her consciousness in confirmation of the testimony of those who saw her.

After this the paroxysms became more frequent, a week seldom passing without her getting up two or three times. Sometimes she did not leave her room, but was occupied in looking over the contents of her trunk, and arranging the different articles of dress. She occasionally placed things where she could not find them when awake, but some circumstances induced the belief that the knowledge of their situation was restored to her in a subsequent paroxysm. In one instance she disposed of her needle-book where she could not afterwards discover it; but after some time had elapsed, she was found one night in her chamber, sewing a ring on the curtain with a needle which she must have procured from the lost book.

The entire paroxism was sometimes passed in bed, where she sung, talked, and repeated passages of poetry. Once she imagined herself at Brattleborough, spoke of scenes and persons with which she was acquainted there, and described the characters of certain individuals with great accuracy and shrewdness, and imitated their actions so exactly as to produce a most comical effect. At this time she denied ever having been at Springfield, nor could she be made to recollet a single individual with whom she was acquainted here, except one or two whom she had known in Brattleborough. Even the name of the people with whom she lived seemed unfamiliar and strange to her.

Generally her conceptions relative to place were, to a certain extent, correct—those relating to time were very commonly inaccurate. She almost invariably supposed it was *day;* hence her common reply when reminded that it was time for her to retire, was, "What! go to bed in the day time?" And when I say her notions relative to place were in accordance with fact, the statement requires considerable limitation. She very frequently imagined herself in a different room from the one where she actually was, and almost always in the room which she usually accupied when awake.

Still her *movements* were always regulated by the senses, and not by her preconceived notions of things. Her chamber was contiguous to a hall, at one extremity of which was the staircase. At the head of the stairs was a door which was usually left open, but which was once closed after she was asleep, and fastened by placing the blade of a knife over the latch. On getting up, she rushed impetuously from her room, and without stopping, reached out her hand before she came to the door, seized the knife, and throwing it indignantly on the floor, exclaimed, "Why do you wish to fasten me in?"

Without entering into a minute detail, I will only mention some of the most remarkable circumstances which occurred at this early period of the complaint.

Allusion has been made to her sewing in the dark, and circumstances render it almost certain that she must at that time have threaded her needle also. Sometime after this occurrence she conceived the plan, during a paroxysm, of making a bag, in which, as she said, to boil some squash. She was then seen to thread a needle in a room in which there was barely light enough to enable others to perceive what she was about, and afterwards, the same night, she was seen to do it with her eyes closed. In this condition she completed the bag, and though a little puckered, as she observed, it still answered very well to boil the squash in.

In one instance she not only arranged the table for a meal, but actually prepared a dinner in the night, with her eyes closed. She first went into the cellar in the dark, procured the vegetables, washed each kind separately, brought in wood and made a fire. While they were being boiled, she completed the arrangement of the table, and then proceeded to try the vegetables to ascertain whether they were sufficiently cooked. After repeated trials, she observed the smallest of them were done—she took them up, and after waiting a little, said the rest would do, and took them up also. They were actually very well cooked. She then remarked that S., a little girl in the family, ate milk, and procured a bowl for her—she also procured one for herself and ate it. As the family did not seat themselves at table, she became impatient, and complained that the men never were ready for their dinner. While engaged in her preparations, she observed a lamp burning in the room, and extinguished it, saying "she did not know why people wished to keep a lamp burning in the day time." On being requested to go to bed, she objected, alleging as a reason, that it was day; but was persuaded to do so by being reminded that she was not well, and that sleep would relieve her head. In the morning she appeared as usual, totally unconscious of the transactions of the preceding night.

At first, the paroxysms occurred only in the night, and generally soon after she went to bed. As the disease advanced, they commenced earlier—she then fell asleep in the evening, sitting in her chair—or rather passed into the state of somnambulism; for her sleep, under these circumstances, was never natural. At a still later period, the attack took place at any hour during the day or evening. After she began to be affected in the day time, the fit seldom commenced when she was in bed; and even when she retired, as she often did, in this state, she usually remained quiet till the paroxysm subsided—though at times she continued to talk and sing. Sometimes she suffered two distinct paroxysms in one day."—pp. 34–41.

The general account here transcribed although sufficient for popular curiosity, is not as minute and scientific as the interests of the profession require; and we shall, therefore, introduce a detailed account of the paroxysms. It relates only to that stage of her disease in which there was extraordinary acuteness of vision.

"*General description of the Paroxysm.*—The state of somnambulism was usually preceded by a full, heavy, unpleasant feeling in the head —sometimes by headach, ringing in the ears, cold extremities, and an irresistible propensity to drowsiness, attended with a feeling as if weights were appended to the eye-lids. There was almost always a slight contraction of the eye-brows, the cheeks were flushed, and sometimes tinged with a crimson hue. By great exertions, the fit might be put off for hours after the appearance of these symptoms; but, in order to gain this reprieve, it was necessary for her to walk, or be engaged in some active employment. The most effectual preventive was exposure to the open air. The moment these precautions were relaxed, and sometimes even in the midst of her active duties, she experienced what she described as a sense of rushing to the head, attended with a loss of the power of speech and motion. If in this state she was immediately carried into the open air, the fit was often arrested; but if this was delayed a moment too long, she lost all recollection, and could not by any efforts be aroused. To a spectator she appeared like a person going quietly to sleep. Her eyes were closed, the respirations become long and deep, her attitude, and the motions of her head, resembled those of a person in a profound slumber. During the fit, the breathing, though sometimes natural, was often hurried, and attended with a peculiar moaning sound, indicative of suffering. At times the pulse was accelerated, but generally it did not vary much from the natural standard. I have remarked, that in her first paroxysm the head was hot, but such was not commonly the ease, nor was there any peculiar throbbing of the temporal arteries—the hands and feet, however, were almost invariably cold.

Her manner differed exceedingly in different paroxysms. Sometimes she engaged in her usual occupations, and then her motions were remarkably quick and impetuous—she moved with astonishing rapidity, and accomplished whatever she attempted with a celerity of which she is utterly incapable in her natural state. She frequently sat in a rocking-chair, at times nodding, and then moving her head from side to side with a kind of nervous uneasiness, the hand and fingers being at the same time affected with a sort of involuntary motion. In the intervals of reading or talking, and even when engaged in these very acts, her nods, the expressions of her countenance, and her apparent insensibility to surrounding objects, forced upon the mind the conviction that she was asleep. Occasionally she was cheerful, disposed to talk, and willing to exercise her powers; the greater part of the time she was irritable and petulant. Pain in a circumscribed spot on the left side of the head, was, I believe, always an attendant on the paroxysm, and frequently occasioned a degree of suffering almost beyond endurance. To this spot she invariably pointed as the seat of her agony when she repeated the expression, "it ought to be cut open, it ought to be cut open." Occasionally the whole system was thrown into agitation, and she presented the appearance of a person in a violent fit of hysterics.

Her eyes were generally closed, but at times they were stretched widely open, and the pupil was then very considerably dilated. These different states of the eye seemed to occasion no difference in the power of seeing—she saw apparently as well when they were closed, as she did when they were open. In the day time she always had the eyes covered with a bandage during the paroxysm, nor would she allow it to be removed for a single moment, unless the room was unusually dark. In order to test the sensibility of the eye, I took one evening a small concave mirror, and held it so that the rays proceeding from a lamp were reflected upon her closed eyelid. When the light was so diffused that the outline of the illuminated space could scarcely be distinguished, it caused, the moment it fell on the eyelid, a shock equal to that produced by an electric battery, followed by the exclamation, "why do you wish to shoot me in the eyes?" This experiment was repeated several times, and was always attended with the same result. It was also tried when she was awake, and the effect, though less striking, was very perceptible. The same degree of light thrown on my eyelids, occasioned no pain.

How far she was sensible to the presence of surrounding objects, it is very difficult to determine; indeed, facts seem to prove that she was not, in every paroxysm, alike in this respect. In the early stage of her complaint, she appeared to take little notice of persons, unless they were connected with her train of thought, and then she regarded those with her only as the representatives of the persons whom she imagined to be present. Nor did the sight or the hearing have any tendency to correct the false impression. Thus, in her first paroxysm, she regarded me as her father, and continued to do so as long as I remained with her; but, in her subsequent fits, this idea was never revived. Her conception of persons was generally made to correspond with the idea of the place in which she conceived herself to be. She was in the habit, when well, of spending her evenings in the room with the children of the family, and it was in their company that she often imagined herself to be during the paroxysm. The questions which were at these times proposed to her to test her powers of vision, were cheerfully and readily answered, because they were questions which it was natural for children to ask; or, at least, she supposed them to proceed from children. Much that she said was also directed to them, though it was evident, at times, her conceptions and perceptions were strangely intermingled. In a paroxysm, soon after the arrival of her father, he asked her a question which she answered by addressing a little boy belonging to the family, who was not then in the room; but his knife which he placed in her hand, she immediately recognized as her father's, and wondered how that came to be in Springfield while he was in Brattleborough. At a later period of her complaint, she appeared to comprehend more of what transpired in her presence, and accordingly she obstinately refused to read cards, or submit to experiments of any kind. These trials she then evidently regarded as so many attempts to impose upon her; and in adopting this conclusion she reasoned with perfect consistency; for if she actually could see as she appeared to—if to her vision, night was converted into day, and darkness into light, while she was unconcious of any thing peculiar to herself, what could be more annoying than to be constantly teased with questions which to her senses were perfectly obvious? If a request were made of her which appeared reasonable, especially if it related to her customary duties, she readily did whatever was required.

There is abundant evidence that she recollected, during a paroxysm, circumstances which occurred in a former attack, though there was no remembrance of them in the interval. A single illustration will suffice, though many more might be given. In a paroxysm, a lady who was present, placed in her hand a bead bag which she had never before seen. She examined it, named the colors, and compared them with those of a bag belonging to a lady in the family. The latter bag being presented to her in a subsequent paroxysm, the recollection of the former was restored—she told the colors of the beads, and made the same remarks respecting the comparative value of the two bags that she had done before. I had taken measures to satisfy myself in the interval that she then remembered nothing of the first impression.

Attempts to rouse her from this state were uniformly unsuccessful. She heard, felt, and saw; but the impressions which she received through the senses had no tendency to waken her. A pailful of cold water was in one instance thrown upon her; she exclaimed, "Why do you wish to drown me!"—went to her chamber, changed her dress, and came down again. Large doses of laudanum were sometimes given her with a view to relieve her pain—it appeared to mitigate her sufferings, and she was observed uniformly to wake soon afterwards.

Excitements of every kind, and particularly attempts to draw forth her peculiar powers, invariably prolonged the fits, and generally aggravated the pain in the head.

At the termination of a paroxysm, she sunk into a profound sleep. The frown disappeared from her brow, the respirations again became long and deep, and the attitude was that of a person in undisturbed slumber. She soon began to gape and rub her eyes, and these motions were repeated after short intervals of repose. In the course of fifteen or twenty minutes from the first appearance of these symptoms, she opened her eyes, when recollection was at once restored. She then invariably reverted to the time and place at which the attack commenced, and in no instance, when under my care, manifested any knowledge of the time which had elapsed, or the circumstances which transpired during the interval.

These paroxysms were very obviously connected with the state of the stomach and digestive organs. Though the appetite was generally good, food often occasioned oppression, and she not unfrequently raised a considerable portion of what she ate. She also had headach, acidity of stomach, and most of the symptoms usually termed dyspeptic. These circumstances had not indeed attracted much attention till after the occurrence of the paroxysms; but I then found that they had existed, in a slight degree, for some time, and that lately her sufferings from this source had been very considerably aggravated. Improper food, and other causes affecting the stomach directly, I am confident, in several instances, occasioned an attack. The very first paroxysm occurred a few hours after she had eaten a large quantity of green currants; and two or three times afterwards, a paroxysm was occasioned by medicine which disturbed the stomach.

During the fit she very often called for food, particularly for apples; but she seldom awoke as soon as usual, after having gratified her appetite. At a time when she had invariably one or two paroxysms daily, I gave her an emetic, and afterwards allowed her to take but a small quantity of the simplest food; under this course she had but one slight attack for five days, and she was in every respect much better. The paroxysm which she had in this instance occurred also under circumstances illustrative of the nature of the complaint. It come on in the stage, when she was on the way to Worcester, and was preceded by sickness, to which she is very subject when riding in a close carriage."—pp. 41–51.

As that part of Miss Rider's case which has seemed the most extraordinary, was her acuteness of vision, we shall transfer to our pages the entire section devoted to that subject.

"*Experiments proving the extraordinary power of vision.*—Though no decisive experiments were at first made to establish the fact, the members of the family in which she lived were very early convinced that she saw both when her eyes were closed, and in the dark. They were irresistably led to this conclusion, when they saw her, night after night, perform that which seemed impossible for her to do without the aid of vision, when at the same time they could discover nothing which indicated the want of sight. She never betrayed any thing like hesitancy or indecision—there was no groping, no feeling after the object which she wished to lay hold of, but the motion was quick and direct, as if perfectly aware of its precise situation. When obstacles were placed in her way, or the position of a thing was changed, she always observed it, and accommodated herself to the change.—This kind of evidence, though perfectly satisfactory to eye witnesses, is not so well calculated to produce conviction in the minds of others as tests of a different kind.

No direct trial of her powers of vision was made until Sabbath evening, Nov. 10th; when it was proposed to ascertain whether she could read with her eyes closed. She was seated in a corner of the room, the lights were placed at a distance from her, and so screened as to leave her in almost entire darkness. In this situation she read with ease a great number of cards which were presented to her, some of which were written with a pencil, and so obscurely, that in a faint light no trace could be discerned by common eyes. She told the date of coins, even when the figures were nearly obliterated. A visitor handed her a letter, with the request that she would read the motto on the seal, which she readily did, although several persons present had been unable to decipher it with the aid of a lamp. The whole of this time the eyes were, to all appearance, perfectly closed.

The second day after this exhibition of her power, she fell asleep in the morning in the act of procuring water from the pump. This was her first attack in the day time. Soon after, on going out of doors, she observed to her companion, "what a beautiful day it is, how bright the sun shines!" It was in fact quite cloudy. When asked by one of the ladies of the family to thread a needle, she refused, saying, "you can do it for yourself." Soon after, she went into a neighboring house, where there was an elderly lady to whom she often rendered this kind of assistance. This lady said, "Jane, I am old, and cannot see very well, will you thread my neadle for me?" She immediately complied with the request, and threaded the needle not only at that time, but once or twice afterwards. She awoke from this paroxysm in the afternoon, and was quite distressed to find the fits beginning to affect her in the day time.

The next morning she fell asleep while I was prescribing for her, and her case having now excited considerable interest, she was visited during that and the following day by probably more than a hundred people. To this circumstance, undoubtedly, is to be attributed the unprecedented length of the paroxysm: for she did not wake till Friday morning, forty-eight hours after the attack. During this time she read a great variety of cards written and presented to her by different individuals, told the time by watches, and wrote short sentences.

For greater security, a second handkerchief was sometimes placed below the one which she wore constantly over her eyes, but apparently without causing any obstruction to the vision. She also repeated with great propriety and distinctness several pieces of poetry, some of which she had learned in childhood, but had forgotten, and others which she had merely read several years since without having ever committed them to memory. In addition to this she sung several songs, such as, "Auld Lang Syne" and "Bruce's Address to his Army," with propriety and correctness. Yet she never learned to sing, and never has been known to sing a tune when awake. She was evidently very much exhausted by these efforts, and at times her sufferings were so extreme that she could not be induced to answer any questions.

On Wednesday, Nov. 20th, I took a large black silk handkerchief, placed between the folds two pieces of cotton batting, and applied it in such a way that the cotton came directly over the eyes, and completely filled the cavity on each side of the nose—the silk was distinctly seen to be in close contact with the skin. Various names were then written on cards, both of persons with whom she was acquainted, and of those who were unknown to her, which she read as soon as they were presented to her. This was done by most of the persons in the room. In reading she always held the paper the right side up, and brought it into the line of vision. The cards were generally placed in her hand for the purpose of attracting her notice, but when her attention was excited she read equally well that which was held before her by another. I do not know that she ever read cards which *she had never seen*, when only the back was presented to her.

Being desirous, if possible, to prove that the eye was actually closed, I took two large wads of cotton, and placed them directly on the closed eyelid, and then bound them on with the handkerchief before used. The cotton filled the cavity under the eyebrow, came down to the middle of the cheek, and was in close contact with the nose. The former experiments were then repeated without any difference in the result. She also took a pencil, and, while rocking in her chair, wrote her own name, each word separately, and dotted the i. Her father, who was present, asked her to write his name. "Shall I write Little Billy or Stiff Billy," was her reply, imagining that the question was proposed by a little boy of the name of William belonging to the family. She wrote *Stiff Billy*—the two words without connection, and after writing them both, she went back and dotted the i in each. She then wrote *Springfied* under them, and after observing it a moment, smilingly remarked that she had left out a letter, and inserted the l in the proper place.

A watch enclosed in a case was handed to her, and she was requested to tell the time—after examining both sides, she opened the case, and then answered the question. Afterwards, but in the same paroxysm, a gentleman present wrote his name in characters so small that no one else could distinguish it at the usual distance from the eye. As soon as the paper was put into her hand, she pronounced the name.—It was thought that any attempt to open the eye would be indicated by the contraction of the skin on the forehead, but though she was closely watched, nothing of the kind was observed.

She also at this time repeated poetry and sung, as before. This she did almost every paroxysm; and though there are some pieces which she must have repeated in this way scores of times, her knowledge of them when she is awake is not in the least improved by the practice. These experiments were performed in the presence of several of the most respectable and intelligent gentlemen in town, and they were all convinced there could be no deception.

While she was in a paroxysm a few evenings afterwards, the lights were removed from her room, and the windows so secured that no object was discernible. Two books were then presented to her which had been selected for the purpose; she immediately told the titles of both, though one of them was a book she had never seen before.

Monday, Nov. 25th, she was removed to my house; but, though she had several paroxysms in the interval, nothing worthy of notice occurred till the 30th. The morning of that day, as she was engaged in her customary employments, she complained suddenly of dizziness, and seated herself in a chair, and immediately became insensible. Soon after, she applied a bandage to the eyes, went to her chamber and changed part of her dress. She then came down, and taking a basket which she had purchased the day before, and which was much soiled, remarked that it was dirty, and she would wash it. This operation she performed with as much neatness and despatch as she could have done when awake.

The room in the front part of the house she had never seen except for a few moments several months since. The shutters were closed, and it was so dark that it was impossible for any one possessing only ordinary powers of vision to distinguish the colors in the carpet. She, however, though her eyes were bandaged, noticed and commented on the various articles of furniture, and pointed out the different colors in the hearth rug. She also took up, and read several cards which were lying on the table. Soon after observing her with a skein of thread in her hand, I offered to hold it for her to wind. She immediately placed it on my hands, and took hold of the end of the thread in a manner which satisfied me she saw it, and completed the operation as skilfully and readily as if she were awake. Having left the room a moment, I found her on my return with her needle threaded, and hemming a cambric handkerchief. She however soon abandoned her work, and was then asked to read a little while aloud. Bryant's Poems were given to her; she opened the book, and turning to the "Thanatopsis," read the whole, (three pages,) and the most of it with great propriety. Something being said about her manner of reading, she observed there were parts of the piece which she did not understand, that she could read it much better if she understood it. The day before, she had procured several *samples* of calico at the shops, portions of some of which had been washed since the commencement of her paroxysm. On their being spread out before her, she not only told the shop at which she obtained each, and named its price, but compared the part which had been washed with the piece from which it was taken, and when there was any change, pointed out the difference.

A colored girl came in and seated herself before her: she was asked if she knew that lady; she smiled and returned no answer. Some one said, "She has a beautiful complexion, has she not?" Jane laughed heartily, and said, "I should think she was somewhat tanned."

At dinner, she took her seat at the table as usual, helped herself to bread when it was offered, presented her tumbler for water, and through the whole time, did not, by her manner or actions, betray the least want of sight. After dinner the bandage which she put over her eyes in the morning, and which she had worn ever since, was taken off, and in its place a black silk handkerchief stuffed with cotton was bound on so as to fit accurately to the nose and cheeks. Though extremely reluctant on account of severe pain in the head, she was at length prevailed on to write a part of the "Snow Storm," one of the pieces which she is in the habit of repeating when asleep. She finished one stanza of six lines, and part of a second. In writing she followed for a time the ruled lines placed under her paper, but they having been displaced, she proceeded without them, continuing, however, nearly in a straight line. In one or two instances she failed to make a proper division of the poetry into lines, and several times misspelled words which she would not have done had she been awake. Twice she noticed the inaccuracy in the spelling, and corrected it at the time, but when writing the same word afterwards she fell into a similar error. A person standing behind her very carefully interposed a piece of brown paper between her eyes and the paper on which she was writing. Whenever this was done she appeared disturbed, and exclaimed, "don't, don't." For sometime I watched her narrowly to ascertain whether the bandage was constantly in place, but I could detect no change in its position.

A watch was presented to her, the face of which was concealed by a piece of brown paper placed between it and the chrystal. Instead of telling the time, she observed, "Any thing but a paper watch!"

In the evening, when the room was so dark that nothing but the position of the windows could be discerned by common eyes, a blue fancy handkerchief was placed before her, and she was asked if she did not wish for a beautiful pink handkerchief—she replied, "I hope I know blue from pink."

The next day, during a paroxysm, she went into a dark room and selected from among several letters, having different directions, the one bearing the name which she was requested to find. She was heard to take up one letter after another and examine it, till she came to the one for which she was in search, when she exclaimed, "Here it is," and brought it out. She also, with her eyes bandaged, wrote of her own accord two stanzas of poetry on a slate; the lines were straight and parallel.

One circumstance I have omitted to mention, which is, the power of imitation which she occasionally exhibits. This extends not only to the manner, but to the language and sentiments of the persons whom she personifies: and her performances in this way are so striking, and her conceptions of character so just, that nothing can be more comical.

This, like her other extraordinary powers, is confined to the somnambulist state—at other times she does not exhibit the slightest trace of it.

Many other circumstances might be added, similar to those which have been detailed; enough, however, has been given to illustrate the peculiar features of this singular case. I have not myself been a witness of every fact here related; but I have mentioned nothing differing in kind or more remarkable in degree than I have seen with my own eyes. However extraordinary these phenomena may appear, therefore, I do not hesitate to vouch for the general accuracy of every statement."—pp. 52—65.

On the 5th of December, six months after the attack, she was removed from Springfield to the hospital at Worcester, and passed from the care of Dr. Belden to that of Dr. Woodward, whose letters to Dr. Belden, make a chapter of the narrative. While in the hospital, she had at one time, a short paroxysm of mania, terminating in hysteria.

The treatment pursued by Dr. Woodward, nearly the same as that recommended by Dr. Belden, consisted in the nitro-muriatic bath to her feet, which were always cold, Calomel in small doses, and the tinctures of guaiacum and sanguinaria as emmenagogues. Her head was shaved and blistered. The cold-dash, when it fell on the "tender spot," made her scream, and restored her to her consciousness. Leeches were also applied to her head. Finally, she took pills of myrrh and iron. Her appepite was voracious and ungovernable. She was always worse for indulging it. During her convalescence in the hospital she was restricted to a low diet. As her paroxysms became slighter, the acuteness of vision decreased. Early in February, 8 months after the attack, she was considered well.

Having presented a history of this case, sufficiently full to place all the important facts before our readers, we shall proceed with our author to one of the speculations which it suggests, to wit—

The increased acuteness of vision. This is the symptom which has excited most astonishment in those who read many of the exaggerated accounts which found their way into the public journals. From these it would appear, that the patient could see through bodies which all the world have pronounced absolutely opake; but this was not the fact. The experiments cited by Dr. Belden show that she could not. The bandages placed over her eyes were not opake, like masks of wood or metal, but still the degree of light which they would permit to pass, must have been so small, that ordinary eyes would scarcely, perhaps, have perceived its presence, and certainly could not have discovered by it the form of any object. We have then, in this case, when divested of all exaggeration, not a little to excite our admiration. Dr. B. supposes that there was not only increased sensibility of the retina, but increased perceptivity of the mind to the luminous impression, arising from a morbid condition of that part of the brain which the phrenologists call the organ of color. She seems, however, to have taken equal cognizance of the outline of objects, and therefore, in phrenological language, the organ of form was not less excited than that of color.

This morbid sensibility of the eye is, as all physicians know, quite a common occurrence. It is generally ascribed to excessive determination of blood to the optic nerves or thalami, and Mr. Stevenson has written a small book to establish this opinion. The two worst cases, however, which have fallen under our own observation, were unattended with inflammatory symptoms, and after resisting a course of antiphlogistic measures, yielded in a great degree to tonics. Both the patients were females. The case of one possesses sufficient interest to justify the introduction of a short history of it in this place.

In the spring of 1825, we were requested to visit Miss W. aged 23, who was represented to labor under an extraordinary sensibility of both eyes to light. She had formerly used her eyes much in straw work and reading, withal leading a most sedentary life, and suffering greatly from constipation. At length her eyes became watery and intolerant of light, though but little inflamed and that at times only. She experienced, however, a great deal of pain in her eye balls, extending back through her head; which was always augmented by exposure of the eye to light, by motion of the arms, by conversation, and by every kind of strong emotion. Her catamenia had been rather in excess. Her tongue was clean, skin cool, and head in no degree too hot.

Blood-letting, cupping, emetics, cathartics and salivation, had mitigated the pain, but left the morbid sensibility of the eyes unabated.

When we saw her, in May, 1825, she had been for several months confined in a dark room, with her eyes bandaged; and she asserted, that even exposing her face to a stream of light, her eyes being well covered, augmented the pain in her eye-balls and their orbits. It appeared, however, that it was chiefly the impress of light upon her mouth and tongue, which gave rise to this effect. In making experiments on this point, we covered her eyes with numerous folds of soft black cloth, pressed firmly upon the cheeks and brows with our own hands. In this situation she could barely tell the difference between the extremes of light and darkness, with her mouth shut; but when she opened it, although not acted on by the rays of the sun direct, she could perceive even slight variations in the quantity of light. So often had this experiment been repeated that the patient and all her friends were entirely convinced of her ability to distinguish between light and darkness by her mouth.

It may not be uninteresting to add, that after the failure of the antiphlogistic course, of which we have spoken, the patient was greatly benefited by drachm doses of rubigo ferri with aloes.

The intelligent reader will perceive that this case presents a fact, which, with several others, goes to show a connexion between the second and fifth pair of nerves. In this young woman the branches of the second and third divisions of the fifth or trifaxcial nerves, spread upon the mucous membrane of the mouth and tongue, appeared manifestly to have acquired a sensibility to light. How far, in Miss Rider's case, they might have acquired the same sensibility, we are unable to say, but believing or inclining to believe, in the possibility of their acquiring such a specific sensibility, we would suggest whether Miss R. did not, in fact, take cognizance of bodies from the action of light upon the facial or lingual extremities of the fifth pair of nerves?

Her aberration of mind, as already intimated, was not reducible to any of the established types of mental disorder. It was *sui generis*, but not unprecedented; as many others, some of whom are mentioned, indeed, by Dr. Belden, have been affected in a similar manner. The chief peculiarity of this case, was its union with such great morbid sensibility of the eyes, a connexion not before observed in the same degree, and which is not necessary to the production of the intellectual phenomena which the case presents.

